# Red Shift, Blue Shift: Investigating Doppler Shifts, Blubber Thickness, and Migration as Explanations of Seasonal Variation in the Tonality of Antarctic Blue Whale Song

**DOI:** 10.1371/journal.pone.0107740

**Published:** 2014-09-17

**Authors:** Brian S. Miller, Russell Leaper, Susannah Calderan, Jason Gedamke

**Affiliations:** 1 Australian Marine Mammal Centre, Australian Antarctic Division, Kingston, Australia; 2 School of Biological Sciences, University of Aberdeen, Aberdeen, United Kingdom; 3 Ocean Acoustics Program, NOAA Fisheries Office of Science and Technology, National Oceanic and Atmospheric Administration, Silver Spring, Maryland, United States of America; Musee National d′Histoire Naturelle, France

## Abstract

The song of Antarctic blue whales (*Balaenoptera musculus intermedia*) comprises repeated, stereotyped, low-frequency calls. Measurements of these calls from recordings spanning many years have revealed a long-term linear decline as well as an intra-annual pattern in tonal frequency. While a number of hypotheses for this long-term decline have been investigated, including changes in population structure, changes in the physical environment, and changes in the behaviour of the whales, there have been relatively few attempts to explain the intra-annual pattern. An additional hypothesis that has not yet been investigated is that differences in the observed frequency from each call are due to the Doppler effect. The assumptions and implications of the Doppler effect on whale song are investigated using 1) vessel-based acoustic recordings of Antarctic blue whales with simultaneous observation of whale movement and 2) long-term acoustic recordings from both the subtropics and Antarctic. Results from vessel-based recordings of Antarctic blue whales indicate that variation in peak-frequency between calls produced by an individual whale was greater than would be expected by the movement of the whale alone. Furthermore, analysis of intra-annual frequency shift at Antarctic recording stations indicates that the Doppler effect is unlikely to fully explain the observations of intra-annual pattern in the frequency of Antarctic blue whale song. However, data do show cyclical changes in frequency in conjunction with season, thus suggesting that there might be a relationship among tonal frequency, body condition, and migration to and from Antarctic feeding grounds.

## Introduction

**Figure 1:**
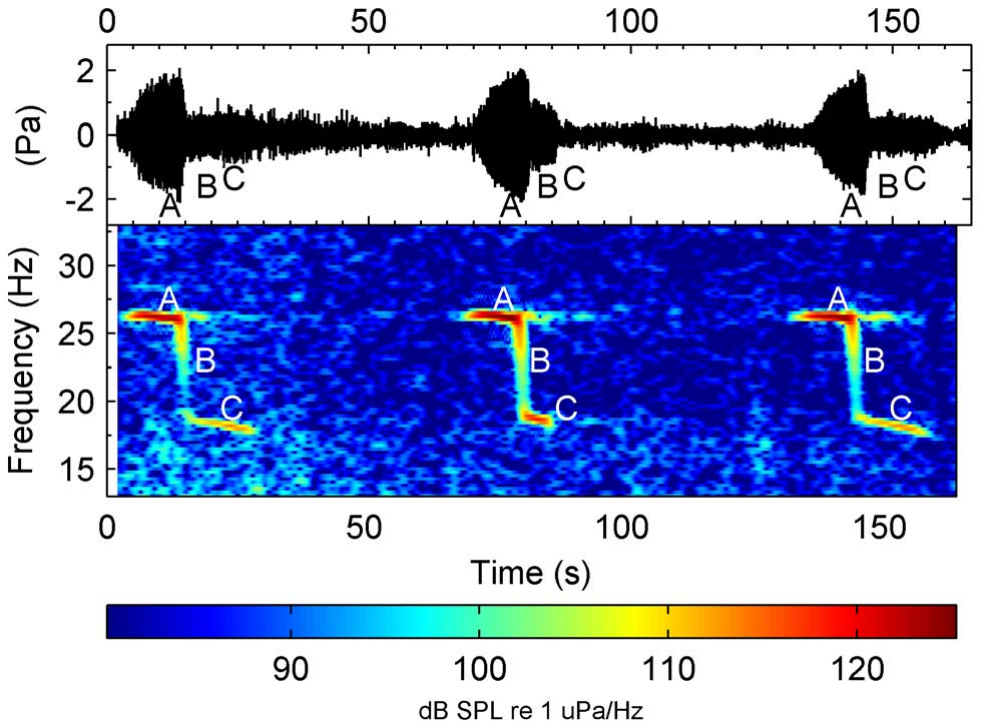
Visualisation of Antarctic blue whale song. Pressure waveform and spectrogram of Antarctic blue whale “z-calls” recorded off Antarctic ice-edge during February 2013. The call is divided into 3 units labelled A, B, and C. Spectrogram was produced using a sample rate of 250 Hz, 1024 point FFT, and 87.5% overlap between time slices. Colors indicate received power spectral density (dB re 1 µPa/Hz).

**Figure 2:**
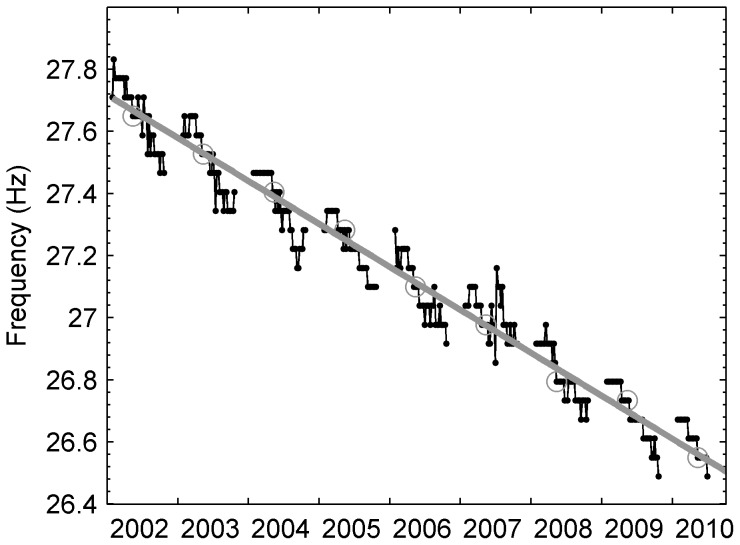
Long-term and intra-annual trends in tonality of Antarctic blue whale song. Long-term trend and intra-annual pattern in tonal frequency of Antarctic blue whale calls. Reprinted with permission from. Gavrilov et al. (2012). Copyright 2012 Journal of the Acoustical Society of America, American Institute of Physics.

Antarctic blue whales (*Balaenoptera musculus intermedia*) produce repeated, stereotyped, low-frequency song comprising three units: an approximately 10 second tonal unit with a frequency of maximum power (henceforth referred to as peak-frequency) around 28–26 Hz and two shorter frequency-modulated downsweeps [1], [2]. In addition to this three-part song, it is believed that Antarctic blue whales also produce songs consisting of only the first tonal unit [2]. The calls of the three-part song have been named ‘z-calls’ because of their characteristic shape when viewed as a spectrogram (Figure 1). Comparison of z-calls recorded in different years has revealed both long-term [3], [4] and seasonal [4] patterns in the tonal frequency of these sounds (Figure 2). Gavrilov et al. [4] reported a linear inter-annual decline of the tonal component of these calls of 0.135 Hz/year (R^2^ = 0.99), and an intra-annual decline between 0.4–0.5 Hz from March to December (R^2^>0.8).

McDonald et al. [3] discussed a number of hypotheses for the long-term inter-annual decline, including changes in population structure, ambient noise, physical environment, and whale behaviour. They concluded that the most likely explanation of the trend was related to increasing population density, and suggested that the tonal decline was an anatomical constraint of the mechanism of sound production that also resulted in a decreased call source level. A key driver of this theory was that the source levels required for whales to keep in acoustic contact with a constant number of conspecifics would not have to be so high if population density were increasing. However, presently there are not enough estimates of the source level of calls (let alone population density) of Antarctic blue whales to test whether source levels have decreased in a manner similar to that predicted by McDonald et al. [3].

Gavrilov et al. [4] proposed that the mechanism behind the intra-annual pattern (Figure 2) might be explained by a gradual decrease in the depth at which songs are produced. They suggested that this decrease in depth could arise from changes in dive behaviour over the length of each season, or that it could be due to other factors such as variations in water temperature or change in blubber mass. However, they considered that such an explanation was not likely to apply to the long-term trend and suggested that changes in whale vocal behaviour remained the most parsimonious explanation for the long-term inter-annual decline.

Here we investigate the Doppler effect [5] as an additional explanation for some of the intra-annual patterns in observations of tonal frequency. Doppler shift is the change in frequency of a wave that arises from relative motion between the source and the receiver of the wave. The equation for Doppler shift can be written as the ratio, *r*, of the measured frequency, *f_m_,* to the true (i.e. non-shifted) frequency *f_w_*:
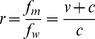
(1)where *v* is the relative speed between the whale and the receiver, and *c* is the speed of sound along the path between source and receiver. If observations are made at a fixed receiver, such as the hydrophone array used by Gavrilov et al. [4], then any potential shift in frequency due to the Doppler effect must arise from movement of the sound source, in this case vocalising Antarctic blue whales.

Seasonal movements of Antarctic blue whales are not well described; however it has been proposed that they, like most baleen whale species, migrate between high latitude summer feeding grounds and low-latitude wintering grounds [6]. There is strong evidence that Antarctic blue whales have a circumpolar Antarctic distribution during the austral summer [7]. In contrast, there are few visual observations of Antarctic blue whales during austral winter [6]. However, acoustic detections of z-calls (distinctive to Antarctic blue whales) provide some of the most compelling evidence that these animals do migrate to mid-or low latitudes in austral winter [4], [8]–[10], despite year-round acoustic detections in the Antarctic [1].

**Figure 3:**
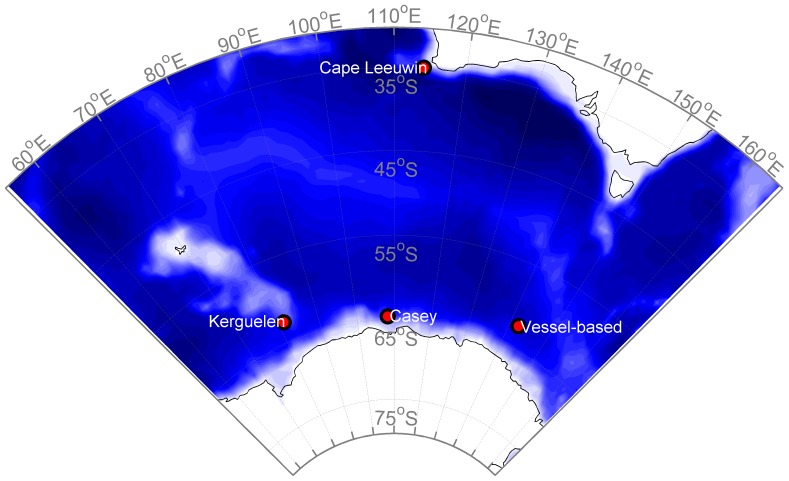
Map of recording sites. Locations of long-term and vessel-based recording stations used in this manuscript for investigation of tonal frequency of the song of Antarctic blue whales. The data from Gavrilov et al. [4] (*i.e.* Figure 2) were observed at Cape Leeuwin.

The temporal aspect of these acoustic detections suggests a mid or low-latitude winter destination for Antarctic blue whales. Stafford et al. [8] reported that low and mid-latitude detections begin in April, and continue through November in the South Pacific, South Atlantic, and Indian Oceans. Samaran et al. [9] found year-round acoustic detections of Antarctic blue whale calls at a mid-latitude site in the Indian Ocean (46°S, 53°E), but proportionally more days with detections in austral winter. Gavrilov et al. [4] also reported near-year round acoustic detection of Antarctic blue whales at Cape Leeuwin, a mid-latitude Indian Ocean site (35°S, 114°E; Figure 3) with detections having highest intensities from May to September.

The peak in intensity in May at Cape Leeuwin could potentially represent the point of closest approach for the majority of the migrating whales, or it could arise from a peak in the number of whales calling. Samaran et al. also found that the month with the highest proportion of days with detected Antarctic blue whale calls off Crozet Island, another mid-latitude location was May [9]. This peak in calling in May at two widely separated locations is further evidence that at this time of year (vocalising) whales are either migrating through to mid-latitudes, calling more frequently, or possibly a combination of the two.

One implication of the Doppler effect could be the ability to track migrating whale populations using recordings made from widely spaced hydrophones located along a latitudinal gradient. For example, at mid latitudes there should be an increase in frequency early in the migration season as the animals approach the hydrophone and a drop late in the season as the animals move away. Such recordings, especially when combined with amplitude information (e.g. [4]), acoustic propagation models (e.g. [11]) and/or acoustic bearings to the sound source [12] could potentially allow for passive acoustic tracking of the migration of populations of vocalizing whales [13].

Here we investigate whether Doppler shift could explain the intra-annual pattern in tonal frequency reported by Gavrilov et al. [4]. We first examine a situation where whale movements were observed and z-calls were recorded simultaneously in order to test whether the Doppler effect on tonal frequencies was measurable for small-scale movements. We then re-examine the intra-annual pattern observed by Gavrilov et al. off Cape Leeuwin [4], and supplement this analysis with year-long recordings from two sites in the Antarctic (Figure 3). Next, we examine whether intra-annual changes in frequency fit with existing knowledge of large-scale migrations of Antarctic blue whales. Additionally, we investigate whether intra-annual variation in tonal frequency is correlated with blubber thickness. Finally, we discuss additional observations and continued data collection that might further test hypotheses to explain the changes in tonal frequency of blue whale song.

## Methods

### A. Vessel-based measurements of frequency and whale speed

During the 2013 Antarctic Blue Whale Voyage of the Southern Ocean Research Partnership, acoustic recordings of Antarctic blue whales were collected along with simultaneous visual tracking [14]. Upon approach, the location of surfacing whales was measured using a video-photogrammetric system (described by Leaper and Gordon [15]) to determine their range and bearing relative to the ship. Acoustic recordings were made during approach using Directional Frequency Analysis and Recording (DIFAR) sonobuoys [16].

The acoustic recording chain was calibrated in accordance with procedures outlined by [12], [17]–[19]. Radio signals from the DIFAR 53D sonobuoy (Ultra Electronics Inc. Canada) were received using an omnidirectional VHF antenna (PCTel Inc. MFB1443; 3 dB gain tuned to 144 MHz centre frequency) and pre-amplifier (Minicircuits Inc. ZX60-33LN-S+) mounted on the mast of the ship at a height of 21 m. The preamplifier was connected to a power splitter via LMR400 cable and signals were received with two WiNRaDiO G39WSBe sonobuoy receivers. Received signals were digitised via a sound board (RME Fireface; RME Inc.), and signals were recorded on a personal computer using the software program PAMGuard [20].

Over the course of the voyage there were dozens of high-quality audio recordings and visual tracks of Antarctic blue whales. However there was only one instance (an encounter on 7 February 2013) of simultaneous video and audio recordings where the whale produced z-calls. This data set was used to investigate whether there was a relationship between whale movements and the received tonal frequency of calls (*i.e.* whether our observations were sensitive enough to detect the Doppler effect). We re-arrange Equation 1 in order to obtain the expected linear relationship between measured tonal frequency, *f_m_*, and velocity yielding:
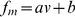
(2)where 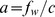 and 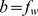. The ‘true’ (*i.e.* non-Doppler shifted) frequency, *f_w_*, was defined to be the long-term trend described by Gavrilov et al., [4]:
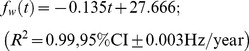
(3)


Here *t* represents the number of years since the start of the dataset: 12 Mar. 2002. It should be noted that the velocity, *v*, corresponds only to the component of movement in the direction of the acoustic wavefront such that:
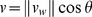
(4)where 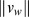 is magnitude of the velocity of the whale, and 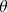 is the difference in angle between the direction of motion of the whale and the bearing from the sonobuoy to the whale.

Locations of Antarctic blue whales obtained via photogrammetric video tracking were assumed to correspond to the “true” location of the whale (at the surface) due to the high accuracy and precision of this technique [15]. Average heading and whale speed were then computed between successive photogrammetric locations. All z-calls in this data set were produced when the whale was out of sight underwater, and linear interpolation between successive photogrammetric locations was used to estimate the locations of the whale at the times when z-calls were received.

Sonobuoys were assumed to drift in a constant direction at a constant speed. The direction and speed of drift were estimated by measuring acoustic bearings to the research vessel (*i.e.* a source with a known location) at intervals of 20 s, and solving for the direction and speed that maximised the likelihood of these measurements [21]. A single estimate of constant drift direction and speed was produced for each sonobuoy for the entire duration of the recording.

Acoustic analysis was restricted to the duration over which there were high-quality photogrammetric measurements. Songs originating from the tracked whale were identified and used for further analysis, while songs that were believed to be from other whales were discarded. Several criteria, including the type of call, temporal pattern of calling, and received level, were used in addition to the acoustic bearing to the source of the song (from the DIFAR sensors) to determine whether or not the call should be included for further analysis.

Measurements of peak-frequency were made from audio recordings of z-calls that were selected for analysis. Peak-frequency measurements were made in the frequency domain by computing the power-spectral density (PSD) for acoustic data spanning the duration of the first tonal unit of the z-calls, which we refer to as unit A. Measurements of peak-frequency were restricted to the band between 25 and 27 Hz in order to exclude potential sources of tonal noise (e.g. engine and/or generator noise from vessels).

The frequency resolution (*i.e.* bin-width) of the PSD is equal to the inverse of the duration of the signal. Due to the relatively short duration of the calls compared to the desired frequency resolution, acoustic waveforms were extended with zeros before the start and after the end of the signal to allow for a sufficiently large number of samples in order to more accurately locate the spectral maxima when computing the spectrum via Fast-Fourier Transform (FFT). Before padding each end with zeros, a Hann window was applied to the acoustic waveform in the time domain in order to minimise any spectral distortion that might arise from the impulsive discontinuity that would otherwise occur at the interface between zeros and acoustic signal.

### B. Long-term measurements of frequency

#### 1. Intra-annual trends in frequency

In contrast to the vessel-based observations, analysis of the intra-annual pattern in frequency relied solely upon the PSD with no attempt to measure individual whale calls. Thus, our analysis methods were identical to those employed by Gavrilov et al., [4]. Measurements of peak-frequency in the Antarctic blue whale band, *f_m_*, were digitized from Figure 5 in Gavrilov et al., [4]. Again, the long-term trend from Gavrilov et al. [4], was taken to be the ‘true’ (i.e. non-shifted) frequency, *f_w_* (equation 3). For each weekly observation reported by Gavrilov et al. [4], the frequency ratio, *r*, of measured frequency to ‘true’ frequency (i.e. the left side of equation 1) was computed. The frequency ratio (i.e. scaling the peak-frequency by the long-term trend) enabled the comparison of intra-annual trends for data that were recorded in different years.

A similar analysis of peak-frequency was also performed on two data sets recorded off Antarctica: data from Acoustic Recording Packages (ARPS; [22]) off Casey Station from 2004 to 2005, and the Kerguelen Plateau from 2005 to 2007. These data were recorded near the sea floor at approximately 1800 m depth at a sample rate of 500 Hz. Before analysis, these data were filtered and re-sampled to 100 Hz in order to maintain a small memory footprint for computations. PSD was averaged daily and the FFT size was 16384 samples to obtain 0.006 Hz frequency resolution; comparable to that of Gavrilov et al. [4]. Portions of the recordings that contained strong broadband noise sources (e.g. large storms) were excluded from the PSD analysis. Additionally long-term spectral averages were visually inspected for time periods when energy from the 20 Hz calls of fin whales was more intense than that of the tonal component of blue whales, and these time periods were also removed. For each daily PSD, the frequency with maximum energy in the 25–29 Hz band was selected as the peak-frequency. Monthly means and standard deviations of these daily peak-frequencies were computed for each station.

All vessel-based work and long-term acoustic recordings were carried out in strict accordance with the approvals and conditions of the Antarctic Animal Ethics Committee for Australian Antarctic Science projects 2683 and 4102. All data used in this work is publicly available via the Australian Antarctic Data Centre (http://data.aad.gov.au/), and are discoverable through the Catalogue of Australian Antarctic and Sub-antarctic Metadata.

#### 2. Doppler effect

In order to assess whether the Doppler effect was a plausible explanation for the intra-annual trends in peak-frequency, we re-arrange equation 1 in order to obtain the relative velocity, 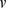, of the source i.e. the population of whales emitting z-calls:
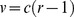
(5)where positive velocities indicate that the direction of travel is towards the observer and negative velocities indicate the direction of travel is away from the observer. The sound speed, *c*, was assumed to be 1500 m/s.

#### 3. Changes in whale anatomy

In addition to Doppler shift, we also conducted a preliminary investigation of the relationship between blubber thickness and the frequency ratio, r. Measurements of the blubber thickness of Antarctic blue whales were digitised from the 1929 Discovery Report by Mackintosh and Wheeler [23]. In accord with the original analysis [23], we considered two size-based groups of Antarctic blue whales: those less than 19 m, and those greater than 23 m. For each size class and we applied weighted least-squares linear regression to investigate potential correlation between the monthly measurements of blubber thickness and the monthly variation in frequency ratio from all recording sites. Monthly variation in frequency ratio, m, was computed as the percentage change in peak-frequency from that of the ‘true’ frequency, m, such that:
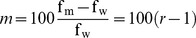
(6)


The variance of *m* was used as the weights when computing the slope and intercept for the weighted least-squares fit.

## Results and Discussion

### A. Vessel-based observations

#### Results

**Figure 4:**
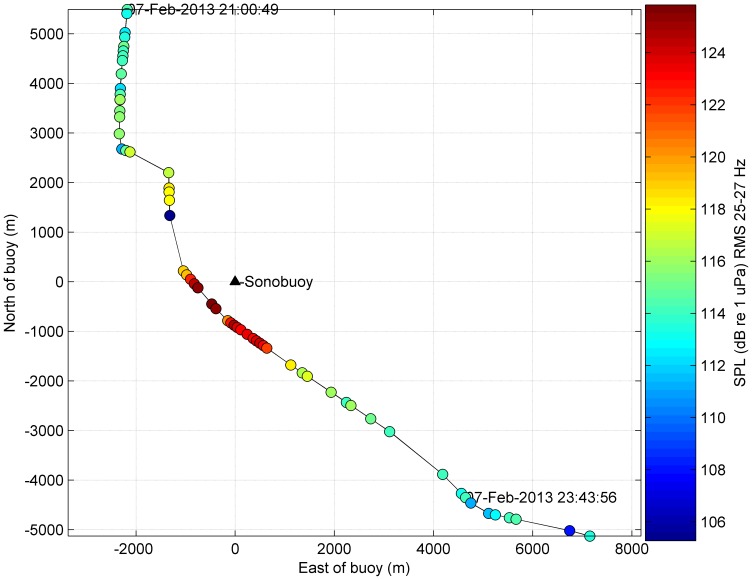
Whale track near a sonobuoy. Whale positions obtained by photogrammetric video tracking (solid black line). All positions are relative to the location of the drifting sonobuoy (black triangle). Filled circles show the estimated location of the whale, relative to the receiver, when z-calls were detected. Color of the circle indicates the received root-mean-square (RMS) sound pressure level (SPL) of call unit A measured in the 25–27 Hz band.

**Figure 5:**
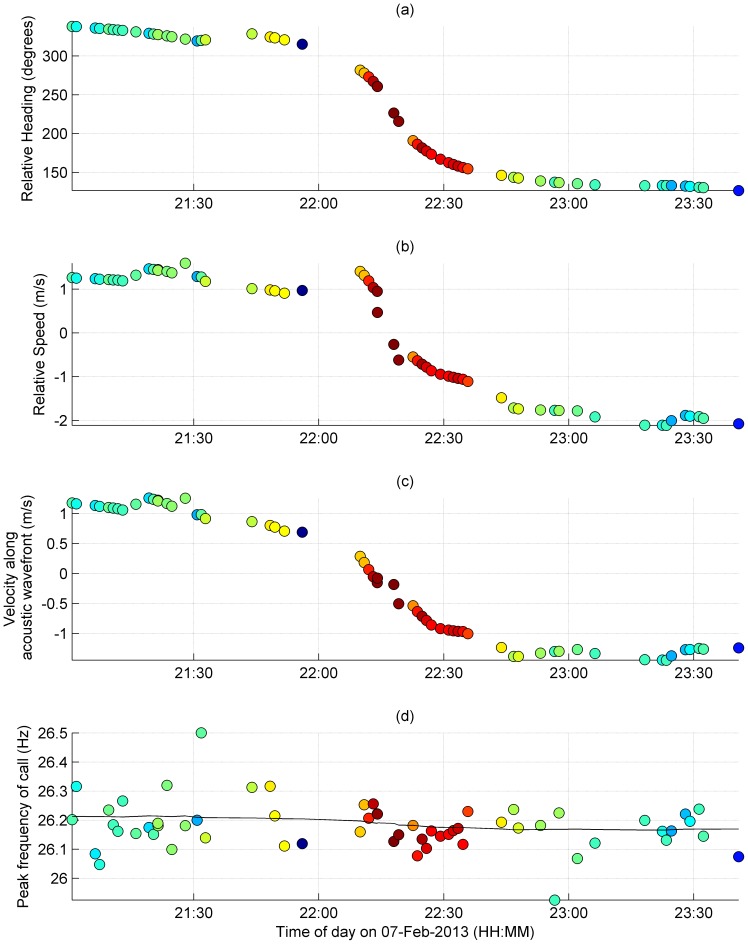
Time series of whale movements. Time series of whale movements shown at the times when z-calls were detected (filled circles). (a) Bearing from sonobuoy to whale. (b) Relative speed between the whale and the buoy. (c) The component of whale velocity in the direction of the acoustic wavefront; (d) Peak-frequency of whale call. The black line in (d) corresponds to the prediction from Equations 2 and 3. Colour of circles corresponds to received level of call as per Figure 4.

During the recording session on 7 February 2013, the whale passed within a kilometre of a sonobuoy (Figure 4). Maximum received levels of whale calls correlated well with the estimated point of closest approach (c. 660 m). This provided confidence that the calls were produced by the photogrammetrically-tracked whale, and that estimates of direction and speed of drift of the sonobuoy (170 degrees; 0.93 m/s respectively) were also consistent. Song was recorded both as the whale was approaching the sonobuoy, and as the whale moved away from the sonobuoy (Figure 5a).

The average speed of the whale between photogrammetrically-derived positions was approximately 2 m/s throughout the encounter. With respect to the buoy, the velocity of the whale ranged from just above 1 m/s to nearly −2 m/s (with negative sign denoting whale movements away from the sonobuoy; Figure 5b). Whale velocity components along the direction of the acoustic wavefront ranged from 1 to −1 m/s (Figure 5c). Measured peak-frequencies ranged between 26.050 and 26.325 Hz, while frequencies predicted from the Doppler effect (equation 2) ranged between 26.160–26.220 Hz, assuming the long-term trend reported by Gavrilov et al. [4] (equation 3).

**Figure 6:**
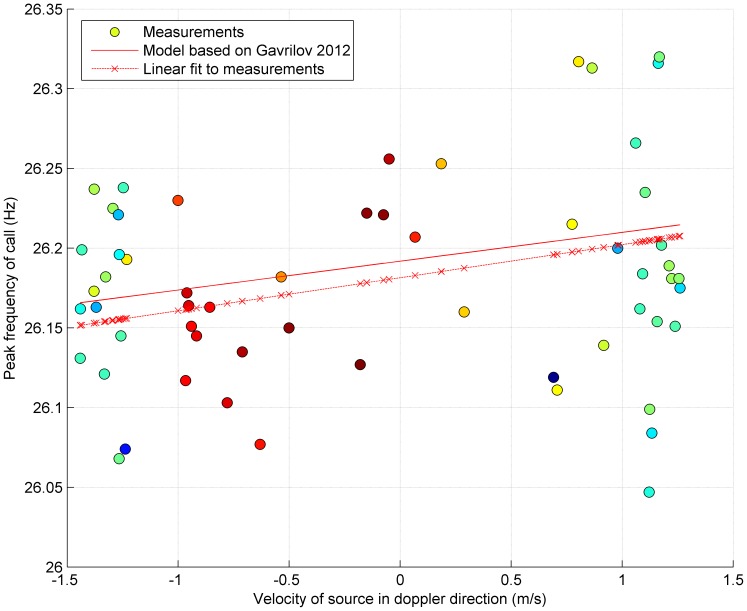
Relationship between observed frequency and movements. Peak-frequency as a function of the velocity of the whale in the direction of the receiver. Filled circles show measured values and colours indicate received level as per Figure 4. Solid line represents the expected frequency shift derived from Equations 2and 3(f_w_ = 26.192; slope = 0.018). Dashed line represents a linear fit to the measurements (f_w_ = 26.182; slope = 0.021; R^2^ = 0.07 (p = 0.039).

The velocity, *v*, explained only a very small proportion of the variability in observed peak-frequency in the multiple calls produced by this individual, *f_m_* (R^2^ = 0.07; p = 0.039; Figure 6). The intercept of the measured peak-frequencies was 26.182 Hz and the standard deviation of the raw data was 0.0814. Applying the Doppler ratio derived from the whale velocity (right-hand side of equation 1), we obtained a base (*i.e.* non-shifted) frequency of 26.181 Hz, and a standard deviation of 0.0784.

#### Discussion

Simultaneous observation of whale movement and acoustic recordings provided an opportunity to test the degree to which the Doppler effect was responsible for frequency variation in calls recorded from an Antarctic blue whale. The observed relationship between speed and peak-frequency (0.021 Hz m^−1^ s) was significant (p = 0.039) and was also very similar to that predicted by the Doppler effect (0.018 Hz m^−1^ s). Furthermore, by ‘correcting’ the raw observations of peak-frequency for Doppler effects, the standard deviation of the data was reduced from 0.0814 to 0.0784 Hz demonstrating that we were able to remove the Doppler effect in order to better estimate the ‘true’ peak-frequency emitted by the whale. However, the variance in measured peak-frequency was greater than would be expected to occur from only Doppler effects due to motion of the whale. This suggests that factors in addition to Doppler shift were responsible for the variation in peak-frequency between independent calls and that these factors dominated the variance.

Change of tonal frequency in blue whale calls may derive from a number of physical factors that are not mutually exclusive. Urick (1983) indicated that both frequency shift and dispersion arise not only from Doppler shift, but also from reverberation of sound as it reflects off the moving sea surface [24]. He further noted that there appeared to be a complex relationship between reverberation, frequency shift, frequency dispersion and wind-speed. Thus whilst the small amount of Doppler shift did undoubtedly occur from the motion of the whale, it appears that it is but one of several factors that contribute to frequency variation between individual calls.

In addition to physical factors in the environment that might have affected the peak-frequency itself, measurement error could also have added to the masking of the contribution of the Doppler effect. Given our careful consideration to use only calls with high-signal-to-noise ratio, the largest source of measurement error is likely to have arisen in estimation of velocities of the whale and sonobuoy. Velocities were estimated by interpolation of surface positions and thus are only an average rather than instantaneous representation of the underwater speed and course of the vocalising whale. Compounding this issue is the fact that the observed swim speeds were all in the same narrow range of approximately 1–2 m/s. Measurement errors in estimating the velocity (of either the whale or sonobuoy) would be expected to increase the deviation of the measured peak-frequency from that predicted by Doppler, but would not necessarily be expected to yield the level of variation observed in the vessel-based measurements. Furthermore, our observed slope of 0.021 Hz m s^−1^ was very similar to that of 0.018 Hz m s^−1^ predicted to arise from Doppler shifts, indicating that measurement errors in both speed and peak-frequency were reasonably small and relatively unbiased.

Lastly, the inherent precision of the whale's sound production was likely a substantial source of variability in peak-frequency. While physical factors and acoustic measurement errors may also contribute to variability, the likelihood that a whale will produce vocalisations which vary in frequency from one call to the next is potentially the largest driver of variation in peak-frequency. While the range of observed peak-frequencies was very small (approximately 0.25 Hz) this range of peak-frequencies is nearly twice as large as the inter-annual decline of 0.135 Hz [4]. Neither the degree to which whales control the pitch of their song (nor the ability of the intended recipient to perceive differences in pitch of said song) have been quantified to date, but further discussion of models of sound production and perception can be found in the following section on whale anatomy and sound production.

Despite these limitations, our results highlight the benefits of combined visual and acoustic observations and demonstrate that we are able to describe the variance in peak-frequency having removed the effect of Doppler shift on the received signals. To our knowledge, the data presented here represent the first successful attempt to measure the Doppler effect in any cetacean vocalisation.

Obtaining more underwater tracks, ideally of higher accuracy and over a wider range of velocities, could help to reduce these confounding effects. Time-depth recorders with yaw-pitch-roll sensors, and acoustic recording capability such as the DTAG or Acousonde could provide one such way to obtain more accurate underwater tracks, and these instruments would also allow comparison of recordings from an instrument moving on the whale with a stationary one. Sonobuoys with integrated GPS receivers and telemetry would also greatly improve the estimation of buoy velocity. Finally, data fusion algorithms could be used to combine position information from video-tracks, DIFAR sonobuoys, acoustic time-depth recording tags, time-differences-of-arrival of sound, and possibly multipath [25], [26] in order to obtain more accurate tracks from the existing and future data sets.

### B. Long-term observations

#### 1. Results

**Figure 7:**
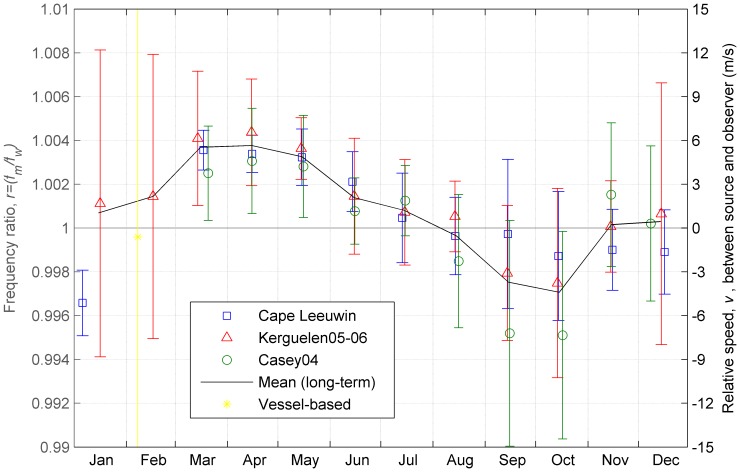
Monthly observations of frequency shift. Markers show the ratio of measured to ‘true’ frequency of Antarctic blue whale song. Measured frequency and ‘true’ frequency are calculated from the data from (Gavrilov et al. 2012) and monthly means are pooled from 9 years of acoustic observations (blue dots). The Antarctic recording stations Kerguelen (red triangle), and Casey (green circle) comprise 2 and 1 years of acoustic observations. Error bars show the monthly standard deviation. The black line connects the monthly mean of all observations from all of the long-term recording stations. The yellow star shows the mean of the vessel-based measurements with error bars denoting one standard deviation (note that error bars for the vessel-based observations extend well beyond the range of the vertical axis for this figure).

The peak-frequencies at each of the long-term recording sites (Figure 3) were compared with the long-term trend in frequency (equation 3) in order to obtain a time series of frequency ratios (i.e. left hand side of equation 1) for each site. Computation of the frequency ratios enabled comparison of the intra-annual trend in frequency among all three sites while accounting for differences caused by the long-term decline in recordings from different years. At all three recording sites the frequency ratios followed the same cyclical pattern over the year, with ratios greater than one more likely to occur from March through June; ratios remaining near one in July and August, and ratios less than one occurring in September and October (Figure 7). The mean annual frequency ratio using measurements from all three sites was 1.0009 with 95% interval between 0.9901 and 1.0077. Mean monthly ratios using data from all sites combined ranged between 0.9971 (October) and 1.0038 (April) (black solid line in Figure 7). Mean monthly ratios and standard deviations showed increased variability compared to the annual mean due to smaller sample size, especially during summer months.

**Figure 8:**
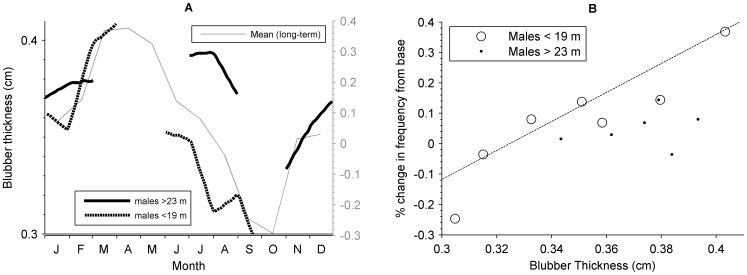
Relationship between blubber and tonal frequency. Seasonal changes around the base frequency measured in this study correlate with seasonal changes in blubber thickness measured by Mackintosh and Wheeler (1929) [23], particularly for males less than 19 m. (**A**) Time series of intra-annual variation in frequency ratio and blubber thickness. Gray line (right vertical axis) represents the monthly change in frequency ratio (equation 6) measured for all recording sites, while black solid and dashed lines are a summary blubber thickness measurements (left vertical axis) digitised from Mackintosh et al., (1929) [23]. (**B**) Relationship between blubber thickness and intra-annual measurements of peak-frequency. Dots represent whales greater than 23 m in length, while open circles represent whales less than 19 m in length again with blubber thickness digitised from Mackintosh et al., (1929). Dashed line shows the least-squares fit at all locations to males less than 19 m weighted by the inverse variance of the monthly frequency ratio (intercept −1.55; slope = 4.77; R^2^ = 0.92; p = 0.004). Males greater than 23 m did not have a significant relationship, so no trend line is shown (intercept = −0.23; slope = 0.69; R^2^ = 0.28; p = 0.59).

Linear regression revealed correlation between the monthly measurements of blubber thickness and the monthly variation in peak-frequency, but only for male blue whales less than 19 m in length (intercept  = −1.55; slope = 4.77; R2 = 0.92; p = 0.004; Figure 8). There was no correlation between blubber thickness and monthly variation in peak-frequency for male blue whales greater than 23 m in length (intercept = −0.23; slope = 0.69; R2 = 0.278; p = 0.594).

#### 2. Intra-annual trend in frequency-ratio

Gavrilov et al., described the intra-annual frequency pattern as declining from March to December and then “resetting” the following March [4]. This sharp “resetting” may have resulted from lack of acoustic observations and measurements at Cape Leeuwin during January and February. By including data from the Kerguelen plateau, we observed a more gradual increase in frequency over January and February that leads to this apparent “reset.” This gradual increase in frequency over the austral summer fleshes out the overall intra-annual pattern with a more sinusoidal rather than sawtooth appearance.

#### 3. Doppler effect

The mean swimming speeds estimated from frequency shift were within the range of plausible speeds for blue whales at all three locations [27]–[30]. However, mean monthly speeds in April and October appear to be too high to be maintained. If they were maintained, then migration would be completed in a matter of days.

Furthermore, the Doppler effect occurs due to the relative speed between the source and the receiver in the direction of the acoustic wavefront, not the absolute speed of the source. This implies that the maximum frequency shifts will occur at the whale's top speed only when the whale's course is directly towards or away from the receiver. If the Doppler effect were responsible for the similar frequency trends in the Antarctic and off Australia, then whales must be simultaneously moving towards both Antarctic and sub-tropical receivers at equal speeds, and this is not consistent with any plausible migration route. Consequently, we believe that it is highly unlikely that the intra-annual pattern in frequency is primarily a result of the Doppler effect during migration.

#### 4. Changes in whale anatomy

After removing the long-term trend, the lowest peak-frequencies produced by whales occurred in October, while the highest occurred in April. The timing of these minima and maxima of peak-frequencies seems to loosely correspond with the arrival to and departure from the Antarctic feeding grounds [6], and thus supports the hypothesis that intra-annual frequency shift may be caused in-part by changes in body condition. Arrival of Antarctic blue whales to the Antarctic feeding grounds is believed to begin in September and increase through December with whales potentially continuing to arrive in the Antarctic into February ([6], [31], W.K. de la Mare unpublished data). Peak-frequency of song also increases from October until March in conjunction with arrival (and presumably feeding) in the Antarctic. By April, most of the whales that will migrate are believed to have departed from the Antarctic [6], and peak-frequency decreases during this time as singers are presumably away from their main feeding grounds.

In addition to the co-occurrence of the extrema of peak-frequency with the arrival and departure of whales to the Antarctic, the gradual variation in mean frequency from month-to-month and the increased variability as whales return to the Antarctic also supports a link between intra-annual frequency patterns and whale anatomy (i.e. body condition). Furthermore, linear regression reveals that the cyclical intra-annual pattern in tonal frequency appears to match that of blubber thickness for male blue whales [23], but only those less than 19 m in length (Figure 8). While there is admittedly a temporal disparity between these two data sets (collected nearly a century apart) and presently a lack of understanding of a causal mechanism linking blubber thickness to tonal frequency, this correlation is intriguing and worthy of further investigation.

#### 5. Sound production, tonal frequency, and intensity

While we cannot rule out a purely behavioural reason for the intra-annual change in frequency, throughout the year the mean variation by month rarely exceeds 0.5% of the “base” frequency for that year. At such low frequencies it is unknown if blue whales, like bottlenose dolphins [32], can perceive a difference in frequency of 0.5% despite indications that they have a hypertrophied cochlea indicative of acute low-frequency hearing [33]. However the change in the mean-monthly peak-frequency throughout the year is less than variation between calls observed during an hour of vessel-based measurements of a single whale. If an individual exhibits this much variability between calls in such a short period of time, it seems unlikely that the observed longer term seasonal pattern of such small shifts in peak-frequency is a result of intentional behavioural changes by all vocalising whales.

In further investigations of intra-annual frequency trends of blue whale song, it may be desirable to consider the intensity (i.e. source levels) of calls and the density of blue whales in addition to the number of calls detected. McDonald et al., proposed that calls with lower peak-frequencies would have lower source levels and should occur when population density is high [3]. Catch data indicates that peak-densitiy of blue whales in the Antarctic occurs in December ([33]; W. K. de la Mare unpublished data) or February [6], thus our observations of lowest peak-frequencies in October, rather than December-February suggest that the intra-annual change in frequency may not necessarily be driven by the same factors that McDonald et al. proposed as the reasons for long-term decline [3].

Sound production in blue whales is not well understood, and initial theories [34], [35] do not appear to satisfactorily describe the mechanism, observed frequency content, and source levels of blue whale sounds [36]. New models of sound production have recently been proposed for mysticetes [36] and tested for humpback whales [37], but remain untested on blue whales. Adam et al. suggest that their model of sound production for humpbacks not only accounts for both the low tonal frequencies, high-source levels, and long duration, but also the high repetition rate of these calls [37]. However, further data on source-levels, density of whales, and whale behaviour (i.e. the purpose of song) would be required to test the hypotheses of Adam et al. [37] and McDonald et al. [3] for Antarctic blue whales.

While we have detailed a clear seasonal pattern in tonal frequency of Antarctic blue whale calls, it remains to be seen whether these intra-annual patterns, like the long-term decline [3], also occur in other populations of blue whales. Although there are hints that similar intra-annual variation in frequency may occur in southeast Indian ocean pygmy blue whales (Balaenoptera musculus brevicauda) [38], further investigation and quantification of these patterns for other populations of blue whales is required. Comparative studies across different populations may yield further insights into the cause(s) of these seasonal variations.

## Conclusions

Variation in the peak-frequency of Antarctic blue whale calls was measured from vessel-based recordings in the Antarctic. This variation was significantly correlated with, but also much greater than, the level that would be predicted by the Doppler effect. This suggests that, at least at low speeds, factors other than the Doppler effect are likely to be the predominant drivers of the seasonal variation in peak-frequency of Antarctic blue whale calls. Furthermore, the fact that the same intra-annual pattern was observed off Cape Leeuwin, Casey Station, and the Kerguelen Plateau makes it unlikely that Doppler shifts coincident with migration are responsible for the intra-annual variation in blue whale peak frequencies. However, this same fact also makes it unlikely that the physical environment (e.g. water temperature, salinity, etc.) is responsible for the pattern, barring extremely long-range acoustic propagation. Thus changes in whale behaviour, or more likely body condition, remain the most parsimonious explanations for the observed intra-annual pattern.

Our results indicate that seasonal patterns in tonal frequency may also yield biological insight into the life-history of Antarctic blue whales complementary to historical [8]–[10], [39], [40] and ongoing [41] studies of the spatial variation and seasonality of acoustic detections. Future studies of intra-annual variation in tonal frequency of blue whale song should consider correcting for Doppler effects, but may only need to do so in situations where whales are moving at high speeds. Further acoustical studies of whale migration should focus on more precise estimates of the number of calling whales, measurements of the intensity (as well as propagation loss and source level of calls) and supplementing acoustical data with anatomical measurements such as length (e.g. [42]–[44]), girth and body condition (e.g. [45]–[47]).
